# Early referral to palliative care in IPF – pitfalls and opportunities in clinical trials

**DOI:** 10.1186/s12931-020-01418-9

**Published:** 2020-07-08

**Authors:** Meena Kalluri, Elisabeth Bendstrup, Kathleen O. Lindell, Giovanni Ferrara

**Affiliations:** 1 Division of Pulmonary Medicine, Department of Medicine, University of Alberta, Alberta Health Services, Edmonton, Canada; 2 Center for Rare Lung Diseases, Department of Respiratory Diseases and Allergy, Aarhus University Hospital, Aarhus, Denmark; 3 Pittsburgh, USA; 4 Division of Pulmonary Medicine, Department of Medicine, University of Alberta, Alberta health Services, Edmonton, AB Canada

**Keywords:** IPF, Palliative care, Early referral to palliative care

**Letter to the editor**

To the Editors,

BMC Respiratory Research

Dear Drs. Bals and Tantisira,

We read with great interest the study by Janssen et al. to assess feasibility of measuring the effect of a palliative care (PC) clinic referral on quality of life, anxiety and depression in  Idiopathic Pulmonary Fibrosis (IPF) [1]. We would like to congratulate the authors for undertaking research in the much-needed area of IPF palliative care. The study was a pilot study and as such not powered to detect impact of intervention. The authors succeeded in demonstrating that this type of study is feasible but the intervention (referral to PC clinic) did not show any impact on health related quality of life (HrQoL) as measured by St. George respiratory Questionnaire (SGRQ) total score, and anxiety and depression (HADS) at 6 months. At 3 months follow up, SGRQ symptoms score and depression showed transient worsening in the intervention group.

In any clinical trial, the goals are to recruit the target population without significant bias, apply standardized interventions and use the best measurement tool to detect the effect of interventions. The authors highlight to several important poinst to consider in understanding the results. First, selection bias may have limited the enrolment into the study. Off 55 eligible patients, only 22 (40%) consented to participate. This is in sharp contrast to recent trials in IPF [2, 3] where all eligible patients meeting inclusion criteria were easily enrolled. The authors correctly note that preconceived ideas of PC may have negatively influenced enrollment. On the other hand, two patients declined to be randomized and wanted to receive palliative care outright. This demonstrates the wide variability in understanding and acceptability of PC within this population. IPF patient and caregiver perceptions of PC are frequently negative at the outset [4, 5]. We believe that this perception, in addition to the design of the study, may have influenced acceptance, adherence to and ultimately success of PC interventions.

In the AmbOx study, 33% of interviewed patients who had negative perceptions of oxygen did not value this therapy and hence chose to discontinue oxygen despite perceived improvement of their symptoms [6]. We suspect this may also be true with PC. If recruited patients have a negative perception of the intervention, it is likely that they may not perceive value despite benefit, and will be less likely to accept and continue therapies, impairing the overall results of the study. While no qualitative analysis was performed in this study, the informal narrative comments do reflect this concern. Some patients found PC beneficial and others did not, these individuals did not value the consultation at all and could not recall the visit. This patient characteristic may have a profound impact on the results and its magnitude outside the intrinsic merits of the intervention itself. Increased understanding of the impact of patient perceptions on acceptance, adherence to interventions and self-reported research outcomes will lead to better study design. It may be important to stratify patients based on this characteristic, a perception and personality informed cohort enrichment [7].

Palliative care is a complex intervention with several different components; where the delivery of each component depends on individual patient’s needs that vary across the disease trajectory [8]. Hence, PC requires individualization, unlike a “pill” and presents challenges to clinical trial design given the heterogeneity in the disease, patient needs and the clinical approach. IPF patients present several unique challenges: some patients and their families’ desire practical advice on how to live well (better treatment of symptoms, improved self-management efficacy etc), others want practitioners to emphasize hope and not just provide information about disease and its management [9]. Some want open and honest discussions on end of life such as how death looks like, how to prepare for the future and more psychosocial support [9–11]. There are even differential information needs between patients and their families based on severity of disease and their own personalities [12, 13]. As patients and families adapt to living with the diagnosis they may move through various phases of shock, denial or acceptance and positive or maladaptive coping [14] depending on their personalities. Future trials will have to consider such adaptive responses that will evolve with disease and time, and design appropriately tailored PC interventions for such a broad range of needs.

How to identify and address these unique needs in clinical practice is the subject of ongoing research. There are several trials underway focused on the various PC components in IPF [15–17]. We hope that these results will highlight the complexity of PC interventions for IPF patients and inform intervention choice in future trials. Specialized research designs such as the sequential multiple assignment-randomized trial (SMART) maybe useful to study such complex PC interventions [18]. Until such time, it may be useful for PC clinical trials to document the actual needs as stated by patients and the individualized interventions applied. Janssen et al. indicate that pharmaceutical interventions were employed in only 2 patients and the majority presumably received education, information or support through the multidisciplinary palliative team. This suggests that most patients’ needs were educational. They do not report if clear practical advice on other aspects of symptom management (e.g. oxygen titrations, pacing, activity modification etc) was delivered. Patients’ needs or goals are not clearly documented at enrolment and therefore, it is uncertain if they were met. In such cases, it is possible that patients may not perceive any tangible benefits as interventions (discussions and actions) were not targeted to needs.

We agree with the authors that discussions regarding prognosis and Advanced Care Planning (ACP) can provoke anxiety if not balanced with practical advice perceived as beneficial and hopeful by the patient. Pooler et al. showed that in the long term, however, ACP and other discussions were perceived to be beneficial by bereaved IPF caregivers. Some subjects reported initial anxiety, but in retrospect, they agreed that it was beneficial to “know” and be prepared for the future (Pooler [19]). While confirmation in prospective studies is needed, it may be important for clinicians to know that short-term anxiety can occur but in the long term, such discussions may have tangible longer-term benefits such as decrease in ineffective and dangerous therapies, and reduced end of life hospitalizations [20]. This poses another problem when designing clinical trials for PC interventions, as the duration of the trial can have a great impact on the measured results and on their interpretation. We also agree that the delivery of such complex PC interventions may need longitudinal support both inside and outside clinics, and involve the care givers [8, 21–24]. A clinic-based intervention while useful may not be able to fully address all needs as disease progresses [9, 23].

Lastly, what is the right measurement tool for quality of life in IPF? HrQoL is a self-report of patient perception of their health status. It is captured by using tools called patient reported outcome measure (PROM). While there are several well validated and frequently used PROM in IPF [25] very few meet all the standards proposed by the Food and Drug Administration (FDA) [26]. The authors used SGRQ in this study; it was originally developed for COPD population, although validated later for IPF. There are items in the SGRQ symptom domain that are not relevant for this disease. Amongst all the available tools, only SGRQ- I and the Kings-brief interstitial lung disease (K-BILD) questionnaire are disease specific and concordant with FDA recommendations for PROM use in clinical trials [27–29]. Even so, K-BILD does not measure cough, a very important and burdensome symptom in this population [30]. This highlights the challenge of finding the right tool to measure HRQol in clinical trials. In the AmbOx study, K-BILD symptom score registered improvement in dyspnea that was not detected by the SGRQ symptom domain. This suggests that the use of disease specific PROM may yield more accurate results and with greater fidelity than a nonspecific PROM. Given the variability in needs based on disease stage, computerized adaptive designs for PROM may be useful rather than using paper based SGRQ that can take approximately 1 h to complete and is also burdensome by patient report in this study. While PROM are essential to measure impact on patients as perceived by them, other outcomes are also equally important when reviewing PC interventions. For example, the authors highlight that one patient was referred to hospice because of the PC referral: This is essential to improve end of life care and hence a vital and important outcome by itself [31].

Future work in this field can build further on Janssen et al’s research. We look forward to a new era of clinical trials where patient centered interventions like palliative care will be assessed in appropriately designed trials, using disease specific PROMs to build the needed evidence base to demonstrate the intrinsic value of palliative medicine for ILD patients.

## Data Availability

Not applicable.

## Abbreviations

IPF: Idiopathic Pulmonary Fibrosis
PC: Palliative care
HrQoL: Health related quality of life
SGRQ: St. George respiratory Questionnaire (SGRQ) total score
HADS: Hospital anxiety and depression questionnaire
AmbOx: Ambulatory oxygen
SMART: Sequential multiple assignment-randomized trial
ACP: Advanced Care Planning
PROM: Patient reported outcomes measure
FDA: Federal Drug Administration
COPD: Chronic obstructive pulmonary disease
SGRQ- I: St. George respiratory Questionnaire for IPF
K-BILD: Kings-brief interstitial lung disease questionnaire
ILD: Interstitial lung disease
